# Identifying Gene Interaction Enrichment for Gene Expression Data

**DOI:** 10.1371/journal.pone.0008064

**Published:** 2009-11-30

**Authors:** Jigang Zhang, Jian Li, Hong-Wen Deng

**Affiliations:** 1 School of Medicine, University of Missouri-Kansas City, Kansas City, Missouri, United States of America; 2 School of Life Science and Technology, Xi'an Jiaotong University, Xi'an, Shaanxi, China; 3 Systematic Biomedicine Research Center, University of Shanghai for Science and Technology, Shanghai, China; Georgia Institute of Technology, United States of America

## Abstract

Gene set analysis allows the inclusion of knowledge from established gene sets, such as gene pathways, and potentially improves the power of detecting differentially expressed genes. However, conventional methods of gene set analysis focus on gene marginal effects in a gene set, and ignore gene interactions which may contribute to complex human diseases. In this study, we propose a method of gene interaction enrichment analysis, which incorporates knowledge of predefined gene sets (e.g. gene pathways) to identify enriched gene interaction effects on a phenotype of interest. In our proposed method, we also discuss the reduction of irrelevant genes and the extraction of a core set of gene interactions for an identified gene set, which contribute to the statistical variation of a phenotype of interest. The utility of our method is demonstrated through analyses on two publicly available microarray datasets. The results show that our method can identify gene sets that show strong gene interaction enrichments. The enriched gene interactions identified by our method may provide clues to new gene regulation mechanisms related to the studied phenotypes. In summary, our method offers a powerful tool for researchers to exhaustively examine the large numbers of gene interactions associated with complex human diseases, and can be a useful complement to classical gene set analyses which only considers single genes in a gene set.

## Introduction

The application of microarray technology has been stimulating methodological development on data analysis that help biologists to gain more insights into biological functions of genes. Conventional statistical analysis methods for gene expression data mainly aim to discover individual genes whose expression changes are associated with a phenotype of interest [1]–[3]. An extension and enhancement to these individual-gene analyses is “gene set analysis”. Gene set analysis utilizes known knowledge of gene sets, such as gene pathways [4], to discover gene sets the expressions of which are associated with a phenotype of interest. Focusing on sets of genes rather than individual genes has at least two benefits: 1) integrating expression changes of genes inside the same gene set can reduces the dimensionality of the dataset and potentially achieve a greater power for detecting differentially expressed genes, even when the expression changes of individual genes are modest; 2) gene set analysis incorporates known biological knowledge. This allows biologists to interpret the microarray data in a manner that is not possible when it is viewed as a collection of individual genes [4] and enhances our ability to understand the functional mechanism that underlies complex human diseases.

A number of gene set analysis methods have been introduced in the last few years [5]–[10]. However, a major challenge for gene set analyses is to discover the interactions among genes, hidden in gene expressions data. Members of a gene set (e.g. a gene pathway) can interact with each other, and these gene interactions can be associated with the phenotype of interest [11]. Previous studies have demonstrated the presence and importance of gene interactions in contributing to complex human diseases [12]–[18]. Thus ignoring gene interactions in gene set analyses can hinder our ability in understanding the gene regulation mechanism underlying human complex diseases.

The purpose of this study is to identify gene interaction enrichments that are associated with a phenotype of interest. We propose a method of gene interaction enrichment analysis in the framework of gene set analysis [8]. We refer to our proposed method as “Interaction-based Gene Set Analysis” (IB-GSA). We apply our method to two publicly available microarray datasets. The results show that our method can identify the gene sets enriched with gene interactions, which conventional methods of gene set analysis ignore or are unable to discover. Identified gene sets and corresponding gene interactions may highlight the underlying gene regulation mechanism that contributes to complex human diseases. Overall, our method provides a complementary approach for identifying gene sets associated with a phenotype of interest, when gene interactions in a gene set are enriched and associated with the studied phenotype.

## Materials and Methods

For simplicity, we focus on two-gene interactions in a microarray experiment with expression profiles from samples in two classes, e.g. presence and absence of a disease. For a gene set *S* (e.g. a gene pathway), assume that in class *k* (*k* = 0 or 1) its gene expression profile consists of *m* genes and *n_k_* samples. These data can be represented by a *m*×*n_k_* matrix ***X***(*S*)*_k_* = (*x_ivk_*) (*i* = 1,…, *m*; *v* = 1,…,*n_k_*), where *x_ivk_* is the gene expression level for the *i*-th gene of the *v*-th individual in class *k*. Let *Y* (*y_vk_* = *k*) be a vector of the phenotypes for samples.

### Gene Interaction Enrichment Analysis

IB-GSA method is to test the null hypothesis that there is no gene interaction enrichment in *S*. When multiple gene sets in a database are evaluated, the estimated significance levels are adjusted for multiple hypothesis testing. Three key steps of our method are outlined as following:

#### Step 1: Measure of gene interaction information

For genes *i* and *j*, neither may have effect on a phenotype of interest. However, when they are jointly considered, they may have a significant effect on the studied phenotype due to the gene-gene interaction. In gene expression data, interaction between gene *i* and gene *j* can be represented by the difference of co-variances or correlations between gene *i* and gene *j* from two different classes.

Prior to performing gene interaction analysis, the expression profile of each gene is standardized by its mean and standard deviation in each class. For example, for gene *i* in the gene set *S*, its expression profile in class *k* is standardized as following:
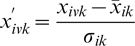
(1)where 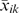 and 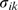 are the mean and standard deviation of expression profile for gene *i* in class *k*. After the standardization, a gene interaction term of genes *i* and *j* 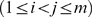 in class *k* can be defined as a cross product of expression profiles of genes *i* and *j* as following [19]:
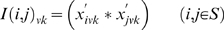
(2)


When the phenotype is binary (i.e., has two classes), whether there is an interaction between gene expression profiles is to test whether the mean cross-products are different across these two classes. Symbolically, this is to test whether 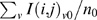 and 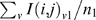 are different. For the case with two genes (gene *i* and *j*), the mean cross-product in a specific class is equivalent to the correlations between the expression levels of the two genes in this class:
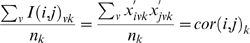



That is, for the case with two genes, the interaction can also tested by comparing the Pearson correlations of expression levels for the two genes in the two classes.

#### Step 2: Calculation of gene set score

We adopt the “maxmean” statistic [8] to calculate a gene set score that reflects the degree of gene interaction enrichment for the gene set *S*. The procedure is briefly described below:

- Typeface="12";Calculate the association between the studied phenotype and each 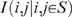 generated from step 1. In this study we use *t*-statistic to test whether there is a difference between 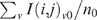 and 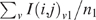, and then transform each *t*-statistic value *t_ij_* to *z_ij_* 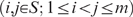. The transformation is 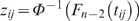, where Φ is the cumulative distribution function (cdf) for a standard normal distribution and *F_n-2_* is the cdf for a *t* distribution with *n*-2 degrees of freedom.
- Typeface="12";Calculate the “maxmean” statistic *T*(*S*) for the gene set *S*, which is defined as:

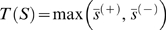
(3)where
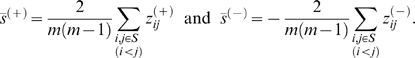



That is, 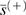 and 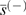 are the averages of the positive and negative *z*-values in the gene set *S*, respectively.

#### Step 3: Permutation test and multiple testing correction for multiple gene sets

To determine if T (*S*) of the gene set *S* is statistically significant, we implement a permutation method, “restandardization”, proposed by Efron and Tibshirani (for details, please refer to reference 8). A large number (*B*) of restandardized permutations are carried out to generate the nominal *p* value for each gene set. In this study we carry out 1,000 restandardized permutations. The empirical *p*-value of the gene set *S* is the fraction of restandardized permutation values T*^B^*(*S*) that exceed (or fall below) the observed value T(*S*):
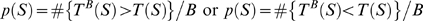
(4)


When multiple gene sets are evaluated, we adjust the estimated significance level to account for multiple hypothesis testing through a standard Benjamini-Hochberg [20] FDR analysis.

### Core Set Extraction for a Significant Gene Set

In reality, when we identify a gene set with enriched gene interactions, it is likely that only a subset of genes in the gene set of interest is associated with the studied phenotype [21], [22]. Thus for each identified gene set with enriched gene interactions, we will extract a core set of gene pairs that chiefly contribute to the statistical variation of a phenotype of interest. The “core set” for the given gene set is a subset that are expected to be more likely associated with the phenotype. Given a statistically significant gene set *S* in gene interaction enrichment analysis, we first calculate the association strength with the phenotype for each gene pair as mentioned above, and then they are sorted in decreasing order of the association strength with the phenotype, *z*_1_≥*z*_2_≥…≥*z_s_*. Select the first *L* genes (*L* = 1,…, *s*) to form a subset *R_L_*. The association statistic with the phenotype for the subset *R_L_* is defined as
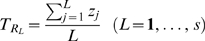
(5)


We define a *p*-value, *p_L_* for each subset *R_L_* based on the permutation of phenotypic data. In this study we carry out 10,000 permutations, and we calculate the *p*-value for the *L*-th subset of the observed data as
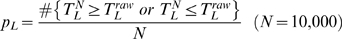
(6)


The “core set” for the given gene set corresponds to the subset with the minimum *p*-value over all the subsets [22].

## Results

In this section, we explore the ability of our method to detect the gene sets in which gene interactions are enriched and associated with a phenotype of interest in two microarray data sets: “p53 cancer data” and “lung cancer data”. The predefined gene sets are obtained from “The Molecular Signatures Database” (MSigDB), which includes 639 sets containing genes whose products are involved in specific metabolic and signaling pathways, as reported in 12 publicly available, manually curated databases. In our analyses, we only use gene sets with at least 15 members observed in microarray data [4]. We perform two types of analyses: 1) detecting the enrichment of gene interactions in gene sets without considering gene marginal effects, as described in the method section and referred as “gene interaction analysis”. 2) detecting the gene marginal effects in gene sets without gene interactions, as proposed by Efron and Tibshirani [8] with the “maxmean” statistic and referred as “main gene analysis”.

### p53 Data Set

The p53 data set contains 50 cell lines. In each cell line, the expression profiles with 10,100 transcripts were obtained after quality control. Out of 50 cell lines, 17 cell lines were classified as normal p53 status while the remaining 33 cell lines carried mutations in the gene of p53. The protein p53 is a transcription factor and acts as a cancer suppressor preventing the development of cancer cells [23]. It regulates genes involved in many key events of cell life such as those regulating cell cycle checkpoints, DNA repair, cell growth, differentiation, apoptosis, and senescence [24].

Results for our analyses are summarized in Table 1. At FDR 0.20 level, in “gene interaction analysis” we identify five gene pathways as significantly associated with p53 mutation status: (i) VEGF signaling pathway; (ii) Gamma hexachlorocyclohexane degradation pathway; (iii) Urea cycle and metabolism of amino groups; (iv) Ether lipid metabolism pathway; and (v) Insulin signaling pathway. These five pathways, however, do not reach the significant level in “main gene analysis”. It suggests that gene regulation patterns of these five pathways may be mainly dependent on the gene interactions (gene correlation changes across two classes), not on the changes of gene expression levels. Thus, when two gene regulation patterns, one mainly dependent on gene interactions and the other on gene expression changes, exist in the p53 dataset, “gene interaction analysis” and “main gene analysis” can complement to each other and give us much biological insights into the genetic regulatory mechanisms of p53 in cancer development.

**Table 1:** Summary of “Gene interaction analysis” and “Main gene analysis” for p53 data set.

Gene interaction analysis		Main gene analysis	
Gene set name	FDR	Gene set name	FDR
VEGF signaling pathway	<0.01	HSP27 pathway	<0.01
Gamma hexachlorocyclohexane degradation pathway	0.09	P53 Hypoxia pathway	<0.01
Urea cycle and metabolism of amino groups pathway	0.09	P53 pathway	<0.01
Ether lipid metabolism pathway	0.09	SA G1 and S phases pathway	<0.01
Insulin signaling pathway	0.15	FMLP pathway	<0.01
		NGF pathway	<0.01
		RAS pathway	<0.01

**Figure 1:**
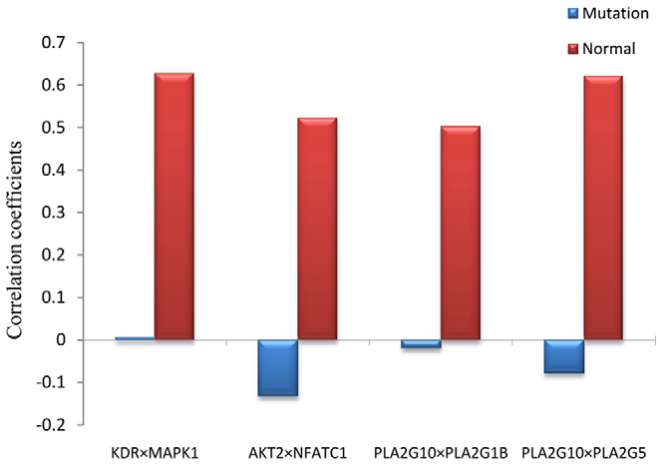
Correlation coefficients of four gene pairs from the VEGF signaling pathway in two different classes.

Taking VEGF signaling pathway as an example, we further extract a core set of gene pairs that chiefly contribute to the variation of p53 status. VEGF signaling pathway is involved in vasculogenesis (e.g. cancer angiogenesis), arteriogenesis, and lymphangiogenesis as well as in both physiological and pathophysiological angiogenesis [25]. By our method, we derive a core set (*p*<1.00e-4) for VEGF signaling pathway, including 187 gene pairs. We illustrate the four gene pairs with top gene interaction effects from VEGF signaling pathway are: 1) KDR and MAPK1; 2) AKT2 and NFATC1; 3) PLA2G10 and PLA2G1B; and 4) PLA2G10 and PLA2G5. As pointed out above, a two-gene interaction effect reflects the change of correlation coefficients of a gene pair in different groups. For the four identified gene pairs, they show strong positive correlations in the normal group, but lower negative or no clear correlation in the mutation group, as shown in Figure 1. For example, for genes KDR and MAPK1, their correlation coefficient is 0.63 in p53 normal group, but 0.01 in the p53 mutation group. Certain genes in these identified gene pairs have been linked to p53 status in the cells by previous studies. For instance, MAPK1 activation has an important role in DNA-damage induced apoptosis. Gene p53 acts as one of the upstream regulators of MAPK1 activation for the induction of apoptosis in cancer cells, and the p53 status can affect the activation MAPK1 [26], [27].

### Lung Cancer Data Set

The lung cancer data set consists of samples from 86 patients, of which 24 were dead and 62 survived. The gene expression profile of each sample contains 5,217 probes. Our analysis results are summarized in Table 2. At FDR 0.20 level, we identify two pathways by “gene interaction analysis”: (i) GSK3 pathway; and (ii) Androgen and estrogen metabolism pathway, which do not reach the significant level in “main gene analysis”, indicating that these two pathways are mainly dependent on two-gene interactions to involve in gene regulation of lung cancer.

**Table 2:** Summary of “Gene interaction analysis” and “Main gene analysis” for lung cancer data.

Gene interaction analysis		Main gene analysis	
Gene set name	FDR	Gene set name	FDR
GSK3 pathway	<0.01	Ceramide pathway	<0.01
Androgen and estrogen metabolism pathway	0.16	AMI pathway	<0.01
		CSK pathway	<0.01

**Figure 2:**
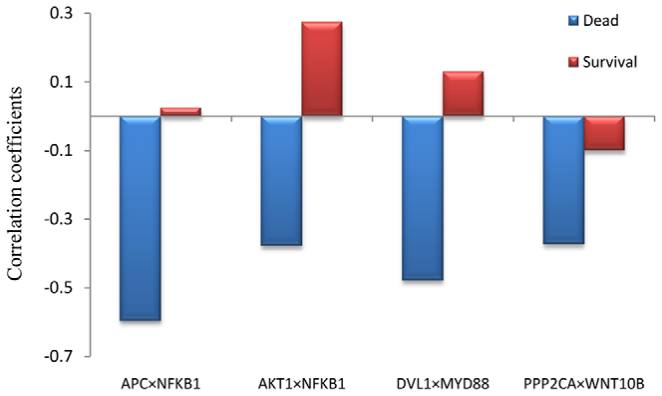
Correlation coefficients of four gene pairs from the GSK3 pathway in two different classes.

To illustrate the effects of gene interactions, we focus on the GSK3 pathway. By our method, we extract a core set (*p*<1.00e-4) for the GSK3 pathway, including 39 gene pairs. The four pairs with top gene interaction effects from it: 1) APC and NFKB1; 2) AKT1 and NFKB1; 3) DVL1 and MYD88; and 4) PPP2CA and WNT10B. As shown in Figure 2, these four gene pairs show negative correlation in the dead group, and lower positive correlation or no clear correlation in the survival group. For example, for genes APC and NFKB1, their correlation is -0.60 in the dead group, but 0.03 in the survival group. Biologically, previous studies have shown that APC is associated with cancer recurrence [28], and AKT1 are associated with several different cancers, such as breast, colorectal, and lung cancers [29]. Thus those gene interactions identified by our method may provide new clue for the gene regulatory mechanisms which are associated with lung cancer.

## Discussion

Although a number of gene set analysis methods have been proposed, they provide little information on gene interactions. However, as gene interactions in a gene set may be associated with the studied phenotype, it is useful for finding potential gene regulation patterns from gene expression data. A computational and statistical challenge in identifying gene interactions in microarray data is that the number of possible gene interactions increases exponentially with the number of genes and that a large number of tests are involved.

In this study, our method not only tests gene interactions in the framework of gene set enrichment analysis, but also extracts core sets of gene interactions that contribute to the variation of a phenotype of interest. More importantly, our method provides a way to integrate these three analyses for identifying target genes and gene interactions related to the trait of interest. Using two publicly available datasets, we have shown how our method can be applied to analyze gene interaction enrichment. The results indicate that our method can discover gene sets with enriched gene interactions hidden in microarray data. In addition, our method is advantageous in that the use of the minimum p-value can reduce the irrelevant gene combinations and extract core sets of gene interactions that chiefly contribute to the statistical variation of a phenotype of interest. Exploration of core sets of gene interactions is a useful step towards further understanding biological mechanisms underlying the gene-set association with the phenotype of interest. The identified gene interactions can be used in the gene regulation construction to investigate a fine structure of the gene regulation patterns that are associated with studied phenotype.

In our method, cross products of gene expression profiles are adopted to identify gene interactions in a gene set. Using cross product term of gene expression profiles, it has two main advantages: 1) this general idea can be extended straightforwardly to test higher-order interaction effects among gene expression profiles for gene expression data. For example, we can test three-gene interactions by using cross-products of three gene expression profiles. Different patterns of gene interactions may produce further insights in the analysis of gene regulation structures; 2) this method can be not only applied to a binary trait, but also a continuous trait for gene interaction analyses. Thus our method provides a general methodology for gene interaction enrichment analysis for gene expression data.

In summary, gene interaction enrichment analysis is a natural exploration step forward for methodologies of gene set analysis. With gene interactions being a basis for the very active field of regulatory network construction [31], [32], our method can give researchers the ability to extract potentially disease-related gene sets and related genes from microarray data, and thus is helpful to delineate the sophisticated knowledge of relevant molecular pathways of disease pathogenesis. Our method can be a useful complement to classical gene set analysis which only considers the single genes in a gene set.

### Web Resources

The URLs for data presented herein are as follows:

P 53 and Lung cancer datasets: http://www.broad.mit.edu/gsea/index.jsp

MSigDB: http://www.broad.mit.edu/gsea/msigdb/index.jsp
